# Topics in Otoplasty: Ear Deformity and Auricular Constriction

**Published:** 2011-07-08

**Authors:** Shawn Diamond, John Reinisch

**Affiliations:** ^a^Division of Plastic and Reconstructive Surgery, Department of Surgery, Weill Cornell Medical College of Cornell University, New York, NY; ^b^Craniofacial and Pediatric Plastic Surgery, Department of Surgery, Cedars-Sinai Medical Center, Beverly Hills, Calif

## DESCRIPTION

The parents of a healthy 4-year-old girl come to the office, complaining that she has “funny looking ears.” They note that the ears have an odd shape since birth. The finding of her routine newborn hearing examination was normal. The patient is taken to the operating room to undergo otoplasty.

## QUESTIONS

1. **What kind of “funny looking ears” does this patient have?**
2. **Describe the epidemiology as well as natural progression of this patient's condition?**
3. **What are the major differences between an ear deformation and an ear malformation? How are these differences reflected embryologically?**
4. **With regard to treatment, what are the common management strategies and/or algorithms?**

## DISCUSSION

This young patient presents with an auricular deformation consisting of low-set, small, and protruding ears. On closer inspection of the anterior surfaces of the ear, there is no delineation of the antihelix from the conchal bowl. Furthermore, the helix appears tight acting like a “purse string”—drawing the upper pole of the ears anteriorly. Taken together, this patient's diagnosis is characteristic of auricular constriction.^1^

 Newborn ear deformations vary in both type and severity along a wide anatomic spectrum. Ultimately, these deformations can produce significant psychological stress including: social withdrawal, depression, and/or lack of companionship in the maturing child—motivating both the parent and the patient to seek correction.^2^,^3^

 Newborn ear deformations occur commonly and have been documented with incidences ranging from 15% to as high as 30% of the United States population.^4^ This may translate into nearly a quarter of a million affected newborns for the year 2011 (CDC).^5^

 Deformations range phenotypically and include the: lop, stahl, constriction, cupping, prominent ear, helical abnormality and nontypical deformations. Studies in Canada have shown that roughly 33% of such deformations spontaneously correct without any intervention.^4^,^6^,^7^ This is confirmed by the adult prevalence of roughly 5%, an obvious decrease from the newborn incidence. However, it is impossible to predict which specific deformation will self-correct.^4^,^6^

 Ear deformations unlike malformations such as Microtia contain all major anatomic components of the adult ear but may be arranged in apparently abnormal and/or nonaesthetic configurations.^1^

 This division between deformation and malformation recalls the embryologic origins of the ear. The ear derives from a fusion of the 3 anterior and 3 posterior hillocks of the first (mandibular) and second (hyoid) brachial arches, respectively, gaining much of its appearance by week 8 of gestation. Impedances in embryological auricular development, thought to be due to stapedial artery malformation and/or impaired flow, produce microtia (ears with missing parts).^1^ However, the underlying causation of auricular deformations (ears with all, but odd parts) is not yet fully understood.

 Management strategy includes early newborn intervention by auricular molding and/or surgery. Correction of ear deformations after the newborn period can only occur successfully through invasive surgical operations. Yet, surgical success is highly linked to patient age at the time of intervention. Auricular cartilage before the age of 6 years is typically highly malleable and long-term results of otoplasty surgery before the age of 6 years result in a 1.9% reoccurrence in the prominent ear spectrum compared to otoplasty after the age of 6 years resulting in a near 30% reoccurrence.^8^ Early surgical intervention is superior, but care must be taken to ensure symmetry given that the adult ear gains 90% of its width and 95% of its height by the age of 10 years.^8^

 A multitude of surgical techniques and algorithms have been developed to address the deformed ear. Techniques such as the Mustarde, Furnas, and Stenstrum cartilage scoring, cartilage removal, and alternatives must be used in an exacting manner to treat the specific deformity; refer to the section on operative procedure.^8^

 Fortunately, the newborn presents with a unique feature that allows for early and nonsurgical treatment of ear deformations, *ear molding*.^4^ Increased levels of estrogen present in the newborn infant produce advantageous pliable effects in auricular cartilage. As levels of estrogen decrease, the cartilage becomes less malleable and more rigid. Thus, if the ear is placed and held in proper anatomic position very early in life, the ear will be “molded” into a more natural shape and fixed over a brief period of time. Yet, molding initiated after 3 weeks of life results in a decrease in success rate by more than 50%.^4^

 Auricular molding produces an improvement in the original deformity and patient satisfaction in more than 90% of cases studied during clinical trial^4^ as well as systematic review.^9^ Therefore, a novel treatment strategy would be to attempt auricular molding on all infants with a deformity, because it is again impossible to identify which ears may or may not ultimately recover naturally. This requires further prospectively designed study.

## OPERATIVE PROCEDURE

**Figure 1:**
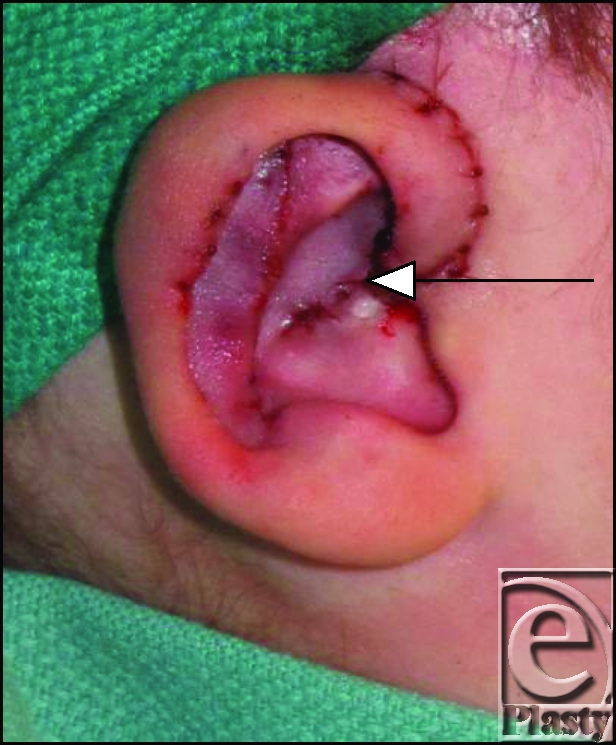
Notice the V-Y incision at the helical root referenced by the arrow.

**Figure 2:**
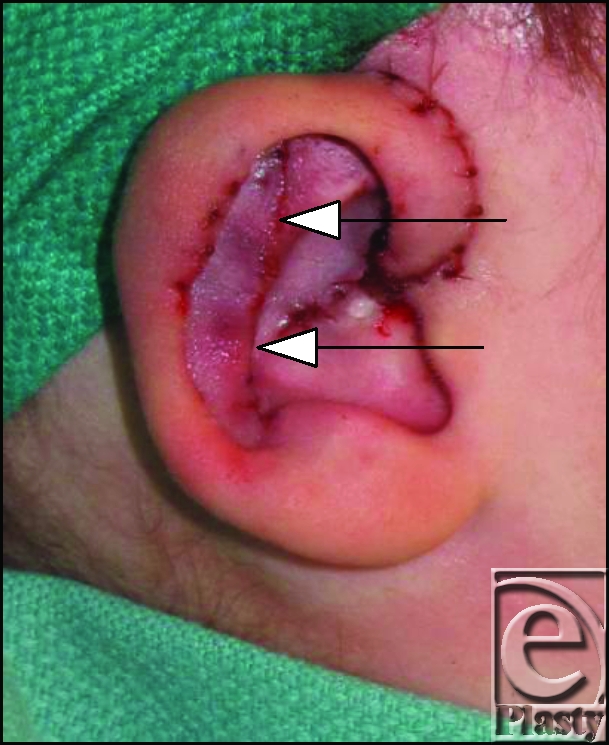
Notice the anteriorly based incision along the antihelical fold with underlying scoring of the cartilage.

After draping the patient in sterile fashion, the more deformed right ear was addressed. A V-Y advancement of the helical root relieved tension and anterior cupping (Fig 1). However, the antihelix was not fully expressed after the advancement. An anteriorly based incision was made along the antihelical curve toward the antitragus with dissection to the perichondrium (Fig 2). This incision was aided by use of hydrodissection via a 22-g needle inserted subcutaneously. A single pass with a 15-blade scalpel was made along the chondral cartilage to score the anterior surface, the Stenstrum technique.^10^ This allowed for adequate reduction of the antihelical fold and draping of the cartilage posteriorly. A single 4-0 nylon Furnas suture was placed from the conchal bowel to the soft triangle to reduce the ear. Additional 4-0 chromic Mustarde mattress sutures were placed along the anterior cartilage to lend support to the antihelix.^8^,^11^,^12^

 The contralateral ear was then addressed for symmetry. The left ear did not require V-Y advancement as helical length was adequate and anterior tension was not significant. The Stenstrum, Furnas, and Mustarde techniques were repeated on the left side. Closure of the skin was performed in the usual fashion with use of interrupted 6-0 Chromic-Gut suture. Wound dressing consisting of Xeroform (Xeroform TM Kendall, Mansfield, MA) and gauze was applied in usual fashion. The patient was asked to follow up within 1 week for routine wound care and cleansing.

 Pre- and postoperative images present on the following page with brief comment.

## Figures and Tables

**Figure:**
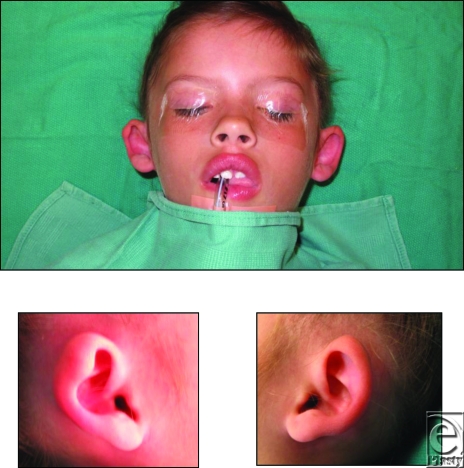


**Figure 3:**
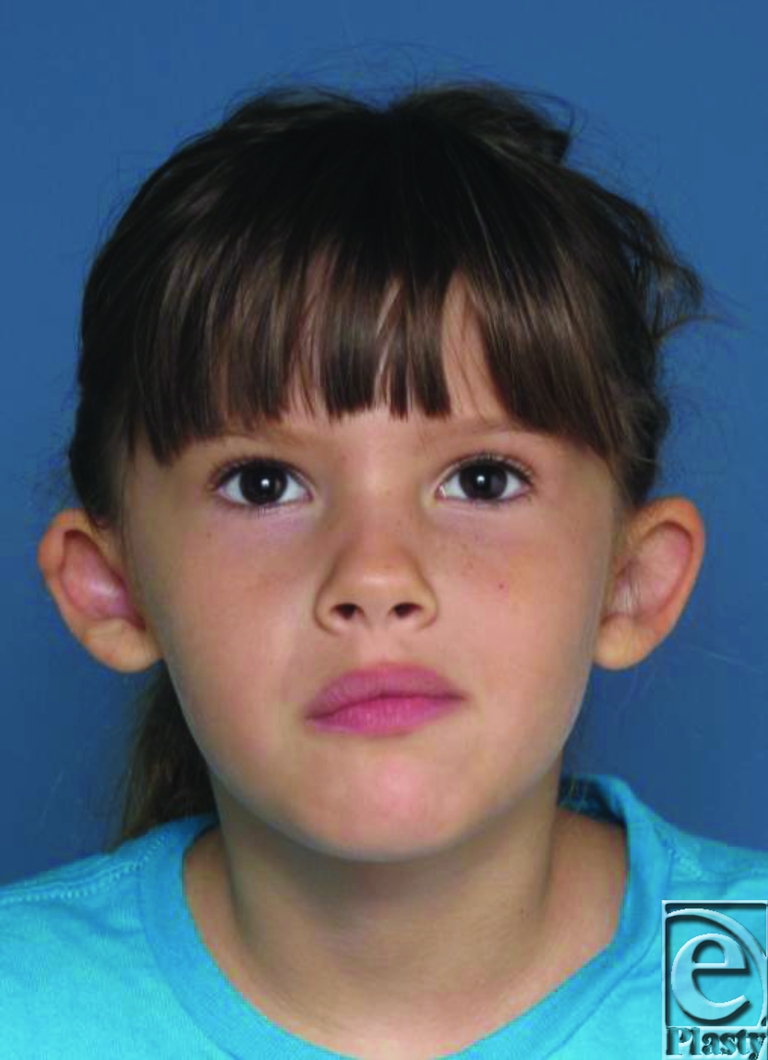
Preoperative Consultation: Note the low-set, protruding quality of the ears without delineation of the antihelical fold and conchal bowl.

**Figure 4:**
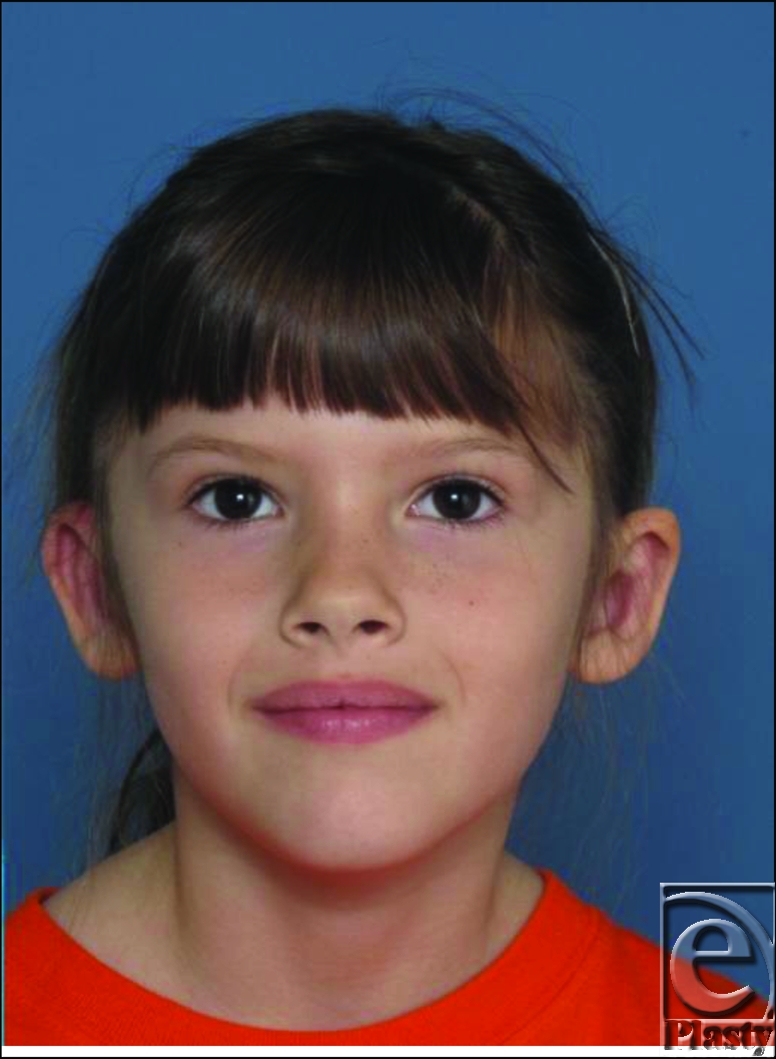
Postoperative visit at 8 weeks: Some erythema and swelling present but markedly improved antihelical/conchal architecture, less protrusion, and opened helix without anterior tension.
